# Welder’s Anthrax: A Review of an Occupational Disease

**DOI:** 10.3390/pathogens11040402

**Published:** 2022-03-26

**Authors:** Marie A. de Perio, Katherine A. Hendricks, Chad H. Dowell, William A. Bower, Nancy C. Burton, Patrick Dawson, Caroline A. Schrodt, Johanna S. Salzer, Chung K. Marston, Karl Feldmann, Alex R. Hoffmaster, James M. Antonini

**Affiliations:** 1 Office of the Director, National Institute for Occupational Safety and Health, Centers for Disease Control and Prevention, Cincinnati, OH 45226, USA; 2 Division of High-Consequence Pathogens and Pathology, National Center for Emerging and Zoonotic Infectious Diseases, Centers for Disease Control and Prevention, Atlanta, GA 30333, USA; kah1@cdc.gov (K.A.H.); wab4@cdc.gov (W.A.B.); cdk5@cdc.gov (C.K.M.); amh9@cdc.gov (A.R.H.); 3 Office of the Director, National Institute for Occupational Safety and Health, Centers for Disease Control and Prevention, Atlanta, GA 30333, USA; crd7@cdc.gov; 4 Division of Field Studies and Engineering, National Institute for Occupational Safety and Health, Centers for Disease Control and Prevention, Cincinnati, OH 45226, USA; njc0@cdc.gov (N.C.B.); ecz4@cdc.gov (K.F.); 5 Office of Science, Centers for Disease Control and Prevention, Atlanta, GA 30333, USA; wpb7@cdc.gov; 6 Division of Healthcare Quality Promotion, National Center for Emerging and Zoonotic Infectious Diseases, Centers for Disease Control and Prevention, Atlanta, GA 30333, USA; pgx7@cdc.gov; 7 Division of Vector-Borne Diseases, National Center for Emerging and Zoonotic Infectious Diseases, Centers for Disease Control and Prevention, Atlanta, GA 30333, USA; hio7@cdc.gov; 8 Health Effects Laboratory Division, National Institute for Occupational Safety and Health, Centers for Disease Control and Prevention, Morgantown, WV 26505, USA; jga6@cdc.gov

**Keywords:** *Bacillus*, welder, welder’s anthrax

## Abstract

Since 1997, nine cases of severe pneumonia, caused by species within the *B. cereus* group and with a presentation similar to that of inhalation anthrax, were reported in seemingly immunocompetent metalworkers, with most being welders. In seven of the cases, isolates were found to harbor a plasmid similar to the *B. anthracis* pXO1 that encodes anthrax toxins. In this paper, we review the literature on the *B. cereus* group spp. pneumonia among welders and other metalworkers, which we term welder’s anthrax. We describe the epidemiology, including more information on two cases of welder’s anthrax in 2020. We also describe the health risks associated with welding, potential mechanisms of infection and pathological damage, prevention measures according to the hierarchy of controls, and clinical and public health considerations. Considering occupational risk factors and controlling exposure to welding fumes and gases among workers, according to the hierarchy of controls, should help prevent disease transmission in the workplace.

## 1. Introduction

The *Bacillus cereus* group classically consists of several *Bacillus* species with closely related phylogeny including *Bacillus anthracis*, *Bacillus cereus*, and *Bacillus thuringiensis*. Recently, the taxonomy of the *B. cereus* group has been updated and expanded based on genomic analysis, which has resulted in the naming of additional species, including *B. tropicus* [1]. *B. anthracis* is the etiologic agent of anthrax, which can manifest as cutaneous, inhalation, injection, or ingestion anthrax, or as primary anthrax meningitis. Genes encoding the major anthrax toxins and the poly-γ-D-glutamic acid capsule are located on two virulence plasmids, pXO1 and pXO2, respectively, and are required for full virulence [2,3,4,5]. *B. cereus* is ubiquitous in the environment and infections are occasionally associated with food-borne illness. Its presence in cultures is often considered to be a contaminant. However, it can cause a variety of infections, e.g., endophthalmitis, bacteremia, cutaneous infection, central nervous system infection, and pneumonia in individuals who have immunocompromising or other underlying conditions or who are recovering from surgery [6]. In patients with *B. cereus* pneumonia, hemoptysis is a common presenting symptom and pulmonary infiltrates are typically present [7,8,9]. Mediastinal widening, which occurs in most cases of inhalation anthrax, has not been observed with these pneumonias [7,8,9].

Both anthrax toxin-producing and non-anthrax-toxin-producing *B. cereus* can cause pneumonia in welders. Since 1997, nine cases of severe pneumonia, caused by species within the *B. cereus* group and with a presentation similar to that of inhalation anthrax, were reported in immunocompetent metalworkers, with most being welders [2,10,11,12,13,14,15,16]. In seven of the cases, isolates were found to harbor a plasmid similar to the *B. anthracis* pXO1 that encodes anthrax toxins [2,11,12,13,14,15,16].

This finding of welders being seemingly disproportionately affected with severe *Bacillus* spp. infections is not limited to *B. cereus* or to recent years. The 1979 anthrax outbreak in Sverdlovsk in the former Union of Soviet Socialist Republics included 77 patients, of which 66 died [17]. The outbreak was thought to be due to the spread of aerosolized *B. anthracis* from a military microbiology facility. Of the 77 patients, 55 were men with a mean age of 42 years. Among the 35 men whose occupations were known, the most common occupation was a welder (n = 7). Few were reported to have had pre-existing medical conditions, but about half were described as moderate or heavy smokers or moderate or heavy drinkers [17]. Cases among welders with underlying pneumoconioses noted at autopsy were more likely to have hemorrhagic pulmonary consolidation than those without such conditions [18].

In this paper, we review the literature on the *B. cereus* group spp. pneumonia among welders and other metalworkers, which we term welder’s anthrax. We describe the epidemiology, including more information on two cases of welder’s anthrax in 2020. We also describe the health risks associated with welding, potential mechanisms of infection and pathological damage, prevention measures according to the hierarchy of controls, and clinical and public health considerations.

## 2. Review of Cases of Welder’s Anthrax

Our case definition for welder’s anthrax comprises an infection caused by an anthrax toxin-expressing species within the *B. cereus* group and manifesting as pneumonia in a metalworker. Seven patients diagnosed with what is now termed welder’s anthrax were reported to the Centers for Disease Control and Prevention (CDC) from 1994–2020 (Table 1). Six were welders and one was another metalworker, and all were confirmed to be infected with *B. cereus* group bacteria containing anthrax toxin genes [2,11,12,13,14,15,16].

Of the six patients with available data on signs and symptoms, over half presented with each of the following: fever or chills, cough, dyspnea, and hemoptysis. All had abnormal chest radiographs and were diagnosed with pneumonia. All were hospitalized and were admitted to the intensive care unit if they survived past the emergency department. Five of the seven patients died [2,11,12,13,14,15,16]. All patients received broad-spectrum antibiotic treatment. One of the surviving patients (Patient F) received raxibacumab, a monoclonal anthrax antitoxin [16].

Of the seven patients, six were male. The median age was 39 years, with a range of 34–56 years. Of the five patients with reported race/ethnicity information, two were white, one was black, and two were Hispanic/Latino. Three had no known co-morbidities or underlying medical conditions. Reported co-morbidities included alcohol use disorder (n = 2), being a current smoker (n = 2), and asthma (n = 1) [2,11,12,13,14,15,16].

Worksites were reported to be in Louisiana for three patients and in Texas for four [2,11,12,13,14,15,16]. Additional work information was sparse in the published case reports. Only two patients (B and G) had information on job tenure (10 and 19 years) [12,16]. Information on the type of welding, job activities, and type of workplace, including indoor or outdoor activities, was not available for most of the patients.

Investigators collected additional work information for Patient F, who worked on the roof of an oil tank located in an oil refinery for 49 days prior to his illness onset. All activities for this project were conducted outside. The patient was part of an eight-person crew, which included three other welders who did not work on the roof of the oil tank. The patient welded on new A36 mild carbon steel using a shielded metal arc welding (or stick) process. Reported electrodes used were 6010, 7018, and 7024. Patient F reportedly wore a 3M 6000 series half-mask respirator equipped with P-100 particulate cartridges while performing welding duties. Patient F performed additional tasks on and around the oil tank, including scrubbing debris off the roof of the oil tank using a wire metal brush and was present during other activities, including sandblasting the paint off the oil tank walls and metal-grinding. However, Patient F reportedly did not use respiratory protection during these non-welding activities. No other crew members were reported to have been ill during this same time period.

Investigators also collected work information for Patient G, who worked as a welder in the wood fabrication shop of a company that manufactures proprietary fixtures for customers in the oil and gas industry. Seven other workers worked in the wood fabrication shop, which had no local exhaust systems but had large bay doors that were usually open. Outside the bay door, on the other side of a paved driveway, was a field with gravel on one side and dirt/grass on the other. Some wood used inside the wood fabrication shop was stored outdoors alongside the gravel field. The patient welded on low-carbon mild steel with no chemical coatings or treatments. The welding process used was Metal Inert Gas (MIG) with solid or flux core wire and 75% argon/25% carbon dioxide shield gas. Patient G welded steel plate end caps to steel tubing in wooden fixtures and performed some pre- and post-welding grinding using a hand-held, AC-powered tool with abrasive disks, and additionally performed flame cutting. The workstation of Patient G was located inside the wood fabrication shop alongside a bay door, and a plasma cutting station was located immediately outside the wood shop on the wall adjacent to the welding station. Compressed air and dry sweeping were routinely used as part of cleanup activities inside the shop. Patient G was reported to have always worn an N95 filtering facepiece respirator and a welding hood when welding but was not fit tested. It is unknown if respiratory protection was used during non-welding activities. It was reported that Patient G and his co-workers ate lunch and took additional breaks outside.

Four case investigations included environmental sampling at the worksite (patients B, C, F, and G), and samples from two investigations yielded *B. cereus* (Table 2) [12,16]. The environmental investigation of patient B’s worksite identified a *B. cereus* isolate from a dust sample that was positive for *B. anthracis* capsule genes. However, it lacked toxin genes and did not genetically match the patient’s clinical isolate [11]. The environmental investigation for patient F identified a bacterial isolate from one soil sample that genetically matched a clinical isolate from the patient [16].

Laboratory testing to detect specific *Bacillus* spp. in environmental samples can be challenging. Its role in epidemiological investigations is limited by its sensitivity; however, focused PCR or culture testing might help confirm a suspected environmental source. A negative result does not necessarily mean that the suspected *Bacillus* spp. strain was not present or was not present in the past.

From 1996–1997, two welders were reported to have rapidly progressive fatal pneumonia caused by *B. cereus* that were not found to have anthrax toxin genes [10]. They did not meet the case definition of welder’s anthrax and were excluded from Table 1. Both patients were otherwise healthy males in their 40s who worked as welders in Louisiana. Additional work information was not reported for either case patient, other than that one patient was exposed to “dust and fumes” at work.

## 3. Welding Processes and Exposures

As of May 2020, nearly 400,000 workers were employed as full-time welders, cutters, solderers, and brazers in the United States, of which only 3.8% were women [19]. Additionally, it is estimated that over 6 million people worldwide have the occupational title of welder either full-time or part-time [20]. Globally, millions of workers not classified as full-time welders may perform welding duties in their jobs, such as shipbuilders, pipefitters, ironworkers, boilermakers, construction workers, farmers, manufacturers, and automotive workers.

Welding provides a powerful industrial tool for the joining of metals. Nearly all metals and alloys can be welded. The American Welding Society has identified over twenty different metal joining processes that are currently being used [21]. Most of these processes are classified under electric arc welding and include shielded manual metal arc welding (or stick welding), gas metal arc welding (or MIG welding), gas tungsten arc welding (or TIG welding), and flux-cored arc welding. Electric arc welding joins pieces of metal that have been made into a liquid by the application of intense heat [22]. The heat needed to melt the metal (>5000 °C) is produced by an electric arc between the work to be welded and an electrode that is continuously fed into the joint. After cooling and solidification, a metallurgic bond is produced [23]. Other types of welding processes include plasma arc welding, submerged arc welding, and oxygas welding.

Electric arc welding produces aerosol by-products composed of a mixture of different metal oxides volatilized from the welding electrode or the flux material incorporated within the electrode [24]. The generated welding fumes are the vaporized metal that has reacted with air to form respirable size particles. The metals most common in welding fumes are iron, chromium, manganese, and nickel. The size distribution of particles generated during electric arc welding has been reported to be multi-modal and dynamically changes with time [24,25]. Three different modes of particle sizes have been observed: (1) nucleation mode (0.01–0.10 mm) of individual primary particles; (2) accumulation mode (0.10–1.0 mm) of agglomerated and coalesced particles formed; and (3) coarse mode (1–20 mm) of non-agglomerated and more spherical particles [24]. In addition, different potentially toxic gases, such as carbon monoxide, ozone, and nitrogen oxides, are commonly generated during electric arc welding.

Each of the welding processes has its own operational and metallurgical advantage, and each may present a different potential health and safety hazard. Due to this, welders are not a homogeneous working group, and their exposure can greatly vary. They work in a variety of settings, such as well-ventilated indoor and outdoor open-air sites (e.g., farms, construction sites, or open-air warehouses) or in confined, poorly ventilated spaces (e.g., ship hulls, boilers, building crawl spaces, underground mines, or pipelines).

The health effects of exposure to welding fumes vary depending on the length and intensity of the exposure and the metals involved. Of particular concern are welding processes involving stainless steel, cadmium- or lead-coated steel, and metals such as manganese, nickel, chrome, zinc, and copper. Fumes from these metals are considerably more toxic than those encountered when welding iron or mild steel. Welding constituents may also interact to produce adverse health effects. Epidemiological studies and case reports of employees exposed to welding emissions have shown an excessive incidence of acute and chronic respiratory disease [26]. These illnesses include metal fume fever, pneumonitis, pulmonary edema, and lung cancer. Exposure to manganese has been associated with Parkinsons-like health effects, such as poor hand-eye coordination, motor slowing, tremor, reduced response speed, mood disturbance, and possible memory and intellectual loss [25,27,28].

Airborne fume concentrations vary greatly in workplaces where welding occurs [29,30,31,32]. Currently, there is no recommended exposure limit (REL) or threshold limit value (TLV) for welding fumes as established by NIOSH and the American Conference of Governmental Industrial Hygienists (ACGIH), respectively. Airborne welding fume concentrations in the workplace are recommended to be kept at the lowest possible levels and to be maintained below exposure limits for the specific metal constituents of the fume that may pose the greatest risk to health (e.g., chromium, nickel, or manganese).

## 4. Possible Mechanisms of Infection and Disease

Several studies have shown an increased risk of pneumonia (defined as bacterial, lobar, and pneumococcal) and mortality among welders and other workers exposed to metal fumes and mineral dusts [33,34,35,36,37,38]. A 2019 review demonstrated that workplace exposures contribute substantially to the burden of community-acquired pneumonia (attributable occupational population fraction, 10%). In seven cohort studies that estimated the risk of pneumonia in welders, metal fumes/welding exposures contributed even more to the burden of community-acquired pneumonia (attributable occupational fraction, 52%) [39]. It was also determined by a scientific panel that the frequency, duration, and severity of upper and lower respiratory tract infections were slightly increased among welders, raising the possibility that exposure to metal fumes might increase susceptibility to lung infection, even with common, relatively harmless infectious agents [40]. An increased mortality from pneumonia among welders has also been reported [41,42].

Evidence suggests that the inhalation of ferrous and other metal fumes in the workplace may predispose workers to lung infections [43]. The mechanisms associated with the immunosuppressive effects of metal fumes after inhalation are mostly unknown. Theories have included that metal fumes (or iron) act as a growth nutrient for bacteria, enhance the binding of bacteria to lung tissues, or impair immune responses in the lung through oxidative stress [33,37,38,42]. Therefore, it is hypothesized that the occupational risk of infection is primarily from occupational exposure to metal fumes. Whether or not occupational activities also result in an increased exposure to these pathogens is not clear. However, the infecting strain of one welder was detected in the environment at his worksite (Table 2).

Animal infectivity studies have indicated that inhalation exposure to common welding fumes during electric arc welding reduced animal body weight and significantly slowed the clearance of a bacterial pathogen after inoculation compared to air controls [44,45]. Bacterial challenge after welding fume exposure in rats resulted in an alteration of multiple cytokines linked to both innate and adaptive immunity. Furthermore, welding fume exposure and the accumulation of metals in the lungs attenuated alveolar macrophage function as they were unable to efficiently respond and clear the bacterial pathogen, resulting in an augmented lung inflammatory response. A graded immunosuppressive response was observed when comparing different welding fumes, with chromium-containing stainless steel welding fumes having the greatest effect on lung defenses against bacterial challenge [46,47].

Like all pathogens, *B. anthracis* and *B. cereus* need iron to survive and thrive, and they have similar, though not identical, mechanisms for its acquisition. They both produce the siderophores petrobactin and bacillobactin. However, the two pathogens have different surface proteins involved in iron uptake: iron-regulated leucine-rich surface protein (IlsA) for *B. cereus* and iron-regulated surface determinant (Isd) proteins for *B. anthracis* [48,49].

Welders may accrue excess iron, become hyperferritinemic, and develop pulmonary siderosis. The appearance of lung opacities on chest x-rays of welders without symptoms of pulmonary illness, a condition now known as siderosis, was reported as early as the 1930s, soon after the introduction of arc welding [50,51]. Siderosis is caused by an excessive accumulation of iron oxide in the lungs, and pulmonary function in welders with siderosis has been observed within normal limits and not different from matched, non-welding controls [52]. A significant portion of iron oxide that is deposited in the lungs after welding fume inhalation is present in alveolar macrophages [53,54] and has been observed to persist in the lungs for years, even after removal from exposure [55].

In a Chinese study, 37 arc welders who had been welding 8 h per day for 2–36 years were compared to sex- and age-matched factory workers with no history of metal exposures. The mean serum iron level in welders was almost twice that of the factory workers (300 ± 137 *vs.* 160 ± 79 µg/L [*p* < 0.01]) [56]. In a study of 241 welders, respirable iron per cubic meter was highly associated (*p* = 0.001) with serum ferritin [57]. Polycythemia and pulmonary siderosis accompanied by fibrosis can also be seen in arc welders [58]. In our review of the five of seven cases of welder’s anthrax from 1994–2020 with reported hematocrits, three patients (Patients B, D, and F) had evidence of iron overload, with hematocrits between 54.5–64.7% [12,13].

Non-occupational risk groups for *B. cereus* pneumonias, though not necessarily caused by toxin-producing *B. cereus* group spp., include other patient groups prone to iron overload: patients with alcohol-use disorders, or acute leukemias [9], and premature infants [6]. In the Sverdlovsk incident, as half the patients with inhalation pneumonia were described as moderate-to-heavy consumers of alcohol, a significant portion of the welders likely also belonged to this subgroup [17]. In our review, Patients F and G were reported to have alcohol use disorders, which might have affected disease severity. In a 2004 NHANES study, mean serum ferritin, transferrin, and serum iron all were increased in mild (120 ng/dL), moderate (151 ng/dL), and heavy (197 ng/dL) consumers of alcohol compared to those who abstained (111 ng/dL) [59]. In a prospective study of 48 patients with acute leukemias or myelodysplastic syndromes, the median serum ferritin was 1549 ng/mL (normal values are 20–250 for males and 10–120 for females). Of these patients with leukemia, 85% had hepatic iron overload, with half of those having severe overload [60]. In one study of premature infants, a fifth had overload, with the most conspicuous association being receipt of multiple transfusions [61].

While iron overload might partially explain the increased susceptibility of welders (and patients with leukemias or alcohol-use disorders or premature infants) for *B. cereus* infections, exposure is still important. A number of authors have noted that soil iron is much higher around welding sites than elsewhere [62,63]. This observation perhaps provides fertile grounds for future research, and measuring soil iron levels may yield useful information.

## 5. Occupational/Public Health Prevention Measures

**Figure 1:**
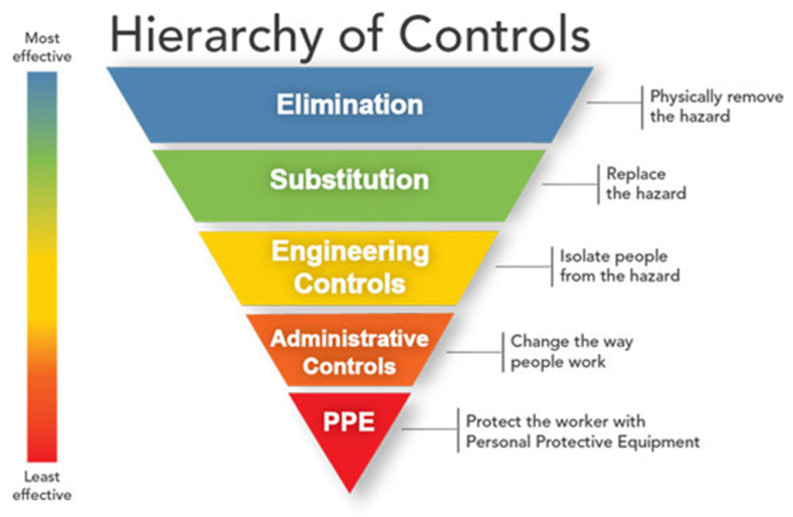
The hierarchy of controls for controlling exposures to occupational hazards. This framework is presented with the methods at the top of graphic as being more effective and protective than those at the bottom. Source: NIOSH.

Occupational health and safety specialists use the hierarchy of controls (Figure 1) to determine how to implement feasible and effective control solutions to occupational hazards [64]. This framework can be used to prevent exposure to welding fumes and gases, and also soils that may be contaminated with opportunistic B. *cereus* group spp. in the workplace. Elimination (removing the hazard) and substitution (replacing the hazard) are the most effective ways to reduce occupational hazards. Engineering controls are physical changes to work processes to remove the hazard or place a barrier between workers and hazards. Administrative controls are methods that change the way the work is performed. Finally, personal protective equipment (PPE) provides a physical barrier between the worker and the hazard. PPE is considered the least effective control measure because it requires a comprehensive program and a high level of worker involvement and commitment for proper use [64].

A key component in occupational safety and health is the workplace hazard assessment, which is a proactive, ongoing process to identify and assess hazards in the workplace [65]. Employers should conduct a hazard assessment on all welders, other metalworkers, and supervisors at worksites [66]. This process involves collecting and reviewing information about the hazards present or likely to be present in the workplace, conducting initial and periodic workplace inspections of the workplace to identify new or recurring hazards, investigating injuries, illnesses, and incidents, determining the severity and likelihood of incidents that could result for each hazard identified, and using this information to prioritize corrective actions.

Employers can then take steps to help reduce exposure to fume and gases from welding and soils that may be contaminated with opportunistic *B. cereus* group spp. during welding operations. Elimination and substitution controls include using a less toxic welding type or consumable and ensuring that welding surfaces are free of any coatings, dirt, and dust that may lead to potentially toxic exposures [66].

Engineering controls can include the use of general and local exhaust ventilation. When welding outdoors or in open areas, it should not be assumed there is adequate general ventilation, even when the welder uses proper positioning and natural drafts. Local exhaust systems should be positioned to draw fume and gases away from the welder and other workers in the area [66].

Administrative controls include maintaining a clean and dirt-free worksite. Workplaces should be routinely cleaned with a vacuum equipped with a high-efficiency particulate air (HEPA) filter or wet cleaning methods. Compressed air and dry sweeping or brushing should not be used. Dust control programs in outdoor workplaces and near workplaces open to the outdoors can minimize dirt and dust exposure, and activities in the immediate vicinity should be limited to help minimize disturbing dry dust. In surrounding areas, adding moisture to roadways and surfaces that are heavily traveled via the application of water, hydroscopic compounds, or surfactants can help control dirt and dust exposures [67]. Water, hydroscopic compounds, and surfactants should not be applied in the immediate area where welding occurs as this may cause an electrocution hazard.

It is essential that welders and other metalworkers understand their potential occupational health risks and how to protect themselves. OSHA’s Hazard Communication Standard requires employers to inform and train workers on potential work hazards and associated safe practices, procedures, and protective measures [68]. Recommended components of a written hazard communication program include educating workers about the health risks from welding and *B. cereus* group spp., signs and symptoms, and how to prevent exposures.

Welders should use PPE such as coveralls and work boots in the workplace to prevent their skin and clothing from being contaminated and taking contaminants home. In addition, use of NIOSH-approved respirators as part of a written respiratory protection program may be needed when other controls do not reduce exposures to safe levels [66,69].

## 6. Clinical Considerations and Medical Countermeasures

In recognition of the association between welding and invasive pneumococcal disease, the 23-valent pneumococcal polysaccharide vaccine has been recommended for welders in the United Kingdom and within a large multi-national corporation for several years [70,71]. Anthrax vaccine adsorbed (AVA) (BioThrax) is licensed for pre-exposure prophylaxis (PrEP) for adults aged 18–65 years at high risk for exposure to *B. anthracis*. AVA induces immunity through the production of antibodies that target the protective antigen component of the anthrax toxins (edema toxin and lethal toxin). The *B. cereus* group strains in the welder-related cases contain the pXO1 virulence factor that produces the anthrax toxins. Since the disease severity seen in these cases was related to the effects of the anthrax toxins, an anthrax vaccine can blunt their effects [72]. When used for PrEP, AVA is administered intramuscularly as a priming series at 0, 1, and 6 months, with booster doses at 12 and 18 months and annually thereafter [73]. Groups considered to be at high risk for exposure to *B. anthracis* include members of the U.S. military deployed to areas designated by the Department of Defense as high risk for exposure, laboratory workers who work with high concentrations of *B. anthracis*, and persons such as farmers, veterinarians, and livestock handlers who might handle animals with anthrax or contaminated animal products [74]. It is currently unknown to what extent environmental species within the *B. cereus* group carry anthrax toxin genes or whether their geographic range extends beyond the U.S. Gulf Coast. Therefore, the role of AVA for PrEP or postexposure prophylaxis (PEP) of welders is not currently recognized or understood. However, for welders working in areas where these infections have occurred, the benefit of the vaccine might outweigh potential adverse events.

Physicians should include anthrax toxin-expressing *B. cereus* group spp. in the differential for welders who present with pneumonia, particularly those working in U.S. Gulf Coast states. Welders and other metalworkers who present with *B. cereus* group spp. infections should be treated in a fashion similar to a patient with inhalation anthrax. Clinical guidelines for the treatment of anthrax are available [75]. Given the severity of these infections, treatment may initially need to be empirical. Patients should receive a minimum of one bactericidal agent plus one protein synthesis inhibitor (e.g., ciprofloxacin and clindamycin) with activity against the *B. cereus* group. However, it should be noted that *B. cereus* has different innate susceptibilities than *B. anthracis* and is usually resistant to penicillins and cephalosporins because of beta lactamase production [6]. If infection with anthrax toxin-expressing *B. cereus* group spp. is suspected, it is important to notify the state health department; a consultation with CDC is recommended. Anthrax antitoxins should be considered as adjunctive therapy if the patient’s clinical condition suggests systemic illness from a *B. cereus* group bacterium. Anthrax antitoxin may be obtained through the U.S. Strategic National Stockpile after consultation with the CDC.

## 7. Public Health Implications

Based on current data collection and surveillance, it is possible that cases of welder’s anthrax were missed due to limited detection and understanding of the pathogen, underdiagnosis, and under-reporting of the patient’s occupation. Discovering risk factors for transmission and assessing hazards in the workplace could help employers plan disease prevention measures according to the hierarchy of controls, such as implementing changes in work practices or an OSHA-compliant respiratory protection program. Including the systematic collection of occupational information as part of infectious disease surveillance might facilitate identifying future workplace-associated cases and outbreaks. Capturing information on both industry and occupation for B. *cereus* group spp. infection cases can further inform public health officials on those specific job risk factors needing further assessment.

To improve data collection in surveillance systems, the NIOSH Surveillance Program at CDC recommends that occupational questions should be standardized, information on both industry and occupation should be collected, and data should be analyzed with standard coding schemes to monitor disease trends in specific industries or occupations and protect workers’ health [76,77]. Other helpful information for the investigation of *B. cereus* group spp. infections includes the employer’s name, work location, job duties, and questions about specific types of welding, metals, and other exposures and protective measures taken.

In addition, employers should provide employee rosters to public health agencies to assist in identifying additional cases when necessary. Employers are currently required to report work-related illnesses resulting in hospitalizations among workers to OSHA programs, and public health agencies should establish agreements with occupational safety and health agencies to share data for surveillance purposes. Outreach in affected areas can prompt healthcare providers to recognize potential work-associated *B. cereus* group spp. infections.

## 8. Conclusions

Welder’s anthrax has emerged as a rare but important occupational infectious disease. Communication and cooperation between clinicians, employers, and public health practitioners is important to identify work-related cases and identify occupational and personal risk factors. More research is needed to better understand the mechanisms of infection and disease among welders. Considering occupational risk factors and controlling exposures to welding fumes and gases among workers according to the hierarchy of controls should help prevent disease transmission in the workplace. Future research is needed to better understand the interplay between exposure to metal fumes and other welding hazards, and the possible increased susceptibility to and severity of lung infection seen in this occupational group. The effectiveness of interventions to minimize workers’ exposure to metal fumes, including engineering controls and respiratory protection, should also be explored.

## Figures and Tables

**Table 1:** Welder’s anthrax cases reported from 1994–2020, n = 7.

Patient	Year of Diagnosis	Age Race/Ethnicity Sex	Occupation	Worksite State	Other Work Information	Co-Morbidities	Anthrax Toxin Genes	Strain	Outcome	Reference
A	1994	42 male *	Welder	LA	None mentioned	None	Yes	*B. cereus* G9241 ^#^	Recovered	[2]
B	2003	39 white male	Welder	TX	Welder for 19 years	Mild asthma, hypertension, hyperlipidemia	Yes	*B. cereus* 03BB87 ^#^	Died	[11,12]
C	2003	56 black male	Metalworker	TX	Worked in foundry, grinding metal for polishing and operating machine	40 pack per year smoker	Yes	*B. cereus* 03BB102	Died	[11,12]
D	2007	47 female *	Welder	LA	Shipyard-related	None	Yes	*B. cereus* LA2007 ^#^	Died	[14,15]
E	2011	39 Hispanic male	Welder	TX	None mentioned	None	Yes	*B. cereus* Elc2	Died	[13]
F	2020	39 white male	Welder	LA	Welded on oil tank on new A36 mild carbon steel using a shielded metal arc welding (or stick) process	Hypertension, gastroesophageal reflux, 25 pack per year smoker, alcohol use disorder	Yes	*B. cereus* LA2020 ^#^	Recovered, received antitoxin	[16]
G	2020	34 Hispanic male	Welder	TX	Worked as a welder for 10 years. Worked in a fabrication shop on low-carbon mild steel using Metal Inert Gas (MIG)	Childhood epilepsy, alcohol use disorder	Yes	*B. cereus* TX2020	Died	[16]

* Unknown race and ethnicity. ^#^ Recent taxonomic updates have subdivided the *B. cereus* group into an additional nine species (https://www.microbiologyresearch.org/content/journal/ijsem/10.1099/ijsem.0.001821), (accessed on 25 March 2022). Whole genome sequence analysis suggests these isolates would be classified as the newly described *Bacillus tropicus*.

**Table 2:** Summary of environmental samples collected at the worksite for four patients with welder’s anthrax.

Patient	Type of Environmental Sample Collected	No. of Environmental Samples Collected	Analytic Method	No. Positive Environmental Samples	Isolate Notes
B [12]	Settled dust and dirt	Not published	Culture	1 *B.cereus* isolate from dust samples from cart	Positive for pXO2 *cap* genes but not anthrax toxin genes; isolate distinct from clinical isolates
C [12]	Settled dust and dirt	Not published	Culture	0	NA
F [16]	Soil, gravel, settled dust and dirt swabs from oil tank *	132	RT-PCR and culture	10 total PCR-positive 4 soil samples from oil tank 4 gravel samples from around worksite 2 swabs from grinder tools and cabinets	One isolate was grown and was a genetic match to the patient’s clinical isolate
G [16]	Soil, settled dust and dirt (sponge and swabs) from surfaces, broom bristles	108	RT-PCR	0	NA

* 53 environmental samples were also collected at and around Patient F’s home. Of those, four samples were positive by RT-PCR, including three swabs from work boots and one swab from a work lunch cooler.

## Data Availability

Not applicable.
